# Body mass index, diabetes, inflammation and survival in COVID-19: a retrospective study evaluating the obesity paradox in an urban hospital center

**DOI:** 10.3389/fcdhc.2026.1946918

**Published:** 2026-10-13

**Authors:** Sanah Sadiq, Rashelle Ripa, Huaqing Zhao, Kristin Criner

**Affiliations:** 1 Department of Endocrinology & Metabolism, Lewis Katz School of Medicine at Temple University Hospital, Philadelphia, PA, United States; 2 Department of Endocrinology, West Virginia University, Morgantown, WV, United States; 3 Department of Biomedical Education and Data Science, Temple University, Philadelphia, PA, United States

**Keywords:** COVID-19, cytokine-storm, diabetes mellitus, glucocorticoids, hyperglycemia, mortality, obesity

## Abstract

**Background:**

Severe coronavirus disease 2019 (COVID-19) is associated with increased morbidity and mortality, particularly among patients with comorbidities such as diabetes and obesity. The combined influence of glycemic control, hyperglycemia, and body mass index (BMI) on disease severity remains incompletely understood, particularly in underserved urban populations.

**Methods:**

This retrospective study evaluated 478 adults hospitalized with confirmed COVID-19 between March and May 2020 at an urban academic medical center to examine the impact of diabetes, glycemic control, and body mass index (BMI) on clinical outcomes. Data collected included demographics, comorbidities, BMI, diabetes type, baseline hemoglobin A1c (HbA1c), inflammatory markers, intensive care unit (ICU) admission, mechanical ventilation, hyperglycemia, and glucocorticoid use.

**Results:**

Among patients with available HbA1c measurements, those with diabetes who developed cytokine storm had significantly higher mean HbA1c than those without cytokine storm. Obesity and overweight were highly prevalent, with 45% of patients classified as obese (BMI ≥30 kg/m²) and 29% classified as overweight (BMI 25–29 kg/m²). Diabetes was associated with increased ICU admission, mechanical ventilation, and steroid-induced hyperglycemia. Mortality was higher among older patients, Black patients, and those with diabetes, hypertension, hyperglycemia, greater glucocorticoid use, or ICU admission. Notably, patients with overweight or obesity demonstrated improved survival compared with those of normal weight, consistent with an obesity paradox in this urban cohort. Multivariable analysis identified steroid-induced hyperglycemia, obstructive sleep apnea, and higher methylprednisolone use as independent predictors of ICU admission, whereas higher BMI was associated with a lower risk of adverse outcomes.

**Conclusions:**

Diabetes and hyperglycemia were associated with greater COVID-19 severity, increased ICU utilization, and higher mortality, while higher BMI offered potential survival benefits. These findings highlight the complex interplay between metabolic dysfunction, obesity, and COVID-19 outcomes, suggesting the presence of an obesity paradox in hospitalized patients, and emphasize the importance of early recognition and management of diabetes and hyperglycemia to improve clinical outcomes.

## 1 Introduction

It is now well known that the coronavirus disease 2019 (COVID-19) causes serious illness and mortality through the unregulated release of inflammatory cytokines that can lead to Acute Respiratory Distress Syndrome (ARDS) and shock (1). Additionally, Diabetes Mellitus (DM) is a well-known risk factor for worse clinical outcomes in patients with COVID-19, including increased disease severity and mortality (2). However, important questions remain regarding the complex interaction between metabolic disease and COVID-19 outcomes. While previous studies have examined the individual effects of obesity and diabetes on COVID-19 severity, fewer studies have evaluated the combined impact of these conditions in patients with concomitant diagnosis of COVID-19 (3). This study aimed to evaluate the prevalence of Type 1 and Type 2 diabetes mellitus, clinical characteristics, hospital outcomes and mortality rates in patients with COVID-19 in an urban population. We hypothesize that patients with diabetes mellitus hospitalized with COVID-19 in an urban center have an increased risk of morbidity and mortality, compared to patients without diabetes, and that obesity would further amplify this risk.

## 2 Material and methods

### 2.1 Research design

A retrospective descriptive analysis was conducted in patients admitted to Temple University Hospital campuses in Philadelphia, Pennsylvania admitted with COVID-19 infection between March 1^st^, 2020 through May 31^st^, 2020, to determine the prevalence of diabetes and effect of the diagnosis of diabetes mellitus on mortality. The study was approved by the Institutional Review Board at Temple University Hospital. Informed consent was waived as data was deidentified and the study met criteria of no more than minimal risk to subjects. The primary outcome was in-hospital mortality, along with length of stay amongst patients hospitalized with COVID-19. Secondary outcomes included DM, hyperglycemia, steroid-induced hyperglycemia, HbA1c, ICU admissions, cytokine storm and endocrine consults.

### 2.2 Study population and data extraction

All patients were between the ages of 18–100 years old and hospitalized between March 1^st^, 2020 through May 31^st^, 2020 with documented COVID-19 infection. The billing code COVID-positive by nasal swab (U07.1) was used to identify patients eligible for study. Charts obtained were independently reviewed by two reviewers to make sure patients met inclusion criteria. Variables were abstracted from the EMR which included: age, gender, body mass index (BMI), race, ethnicity; diagnoses of Type 1 diabetes mellitus or Type 2 diabetes mellitus, Diabetic Ketoacidosis (DKA), post-transplant DM, hyperglycemia, steroid-induced hyperglycemia, COVID-19 by nasal swab, sleep apnea, hypertension, Chronic Kidney Disease (CKD), lung disease, heart failure, obesity, renal failure on dialysis. Lab values included Hemoglobin A1c (HbA1c), initial capillary glucose values, hemoglobin and hematocrit, Glomerular Filtration Rate (GFR, mL/min/1.73 m²), liver enzymes, triglyceride levels, inflammatory markers (D-Dimer, ferritin, IL-6, creatinine kinase, prothrombin time). Other variables collected included use of medications for diabetes (insulin, Dipeptidyl peptidase-4 inhibitors (DPP4 inhibitors), metformin), glucocorticoid dosages and duration, length of stay in days, ICU admissions, mechanical ventilation and endocrinology consult (**Supplementary Tables 1–6**). Diabetes mellitus was defined based on documentation in the medical record, including the problem list, provider notes, use of diabetes-specific medications, along with an HbA1c ≥ 6.5%. New-onset diabetes was defined as an HbA1c ≥6.5% or persistent hyperglycemia without a prior diabetes diagnosis. Hyperglycemia was defined as any blood glucose value ≥ 200 mg/dL (> 11.1 mmol/L) on at least two occasions during the hospitalization. Steroid-induced hyperglycemia was defined as new-onset hyperglycemia occurring after the initiation of systemic glucocorticoids. Obesity was defined as a BMI ≥ 30 kg/m² and other comorbidities were identified through review of provider notes, problem lists, and medication records. COVID-19 infection was confirmed by a positive SARS-CoV-2 PCR test from a nasopharyngeal swab or, in cases of negative PCR testing, by imaging findings on chest CT consistent with COVID-19 (**Supplementary Tables 7, 8**). Disease severity was determined using clinical features, including symptoms, radiographic findings on chest X-ray (CXR) or high-resolution computed tomography (HRCT), respiratory support requirements, and hemodynamic status (**Table 1**). ICU admission was defined as any stay in an intensive care unit during hospitalization and mechanical ventilation included any invasive ventilatory support. Deidentified data was stored on an excel spreadsheet. Missing information and information that could not be automatically abstracted was entered manually after chart review.

**Table 1:** COVID-19 severity stage definitions.

Stage	COVID-19 status	Presence and level of SX	SX type	CXR/HRCT	Respiratory support	Hemodynamic support	Triage
0	+	None	None	Normal	None	None	Home
I	+	Mild	Fever < 101°F, sore throat, nasal congestion, cough, chest tightness, myalgia, body ache, fatigue, diarrhea	None	NC ≤ 3 L O_2_ at rest	None	Home
II	+	Moderate	SOB, RR 20–25, HR ≤ 110 bpm, fever ≤ 101°F	GGO or peripheral consolidation at lobar level	NC ≥ 4 L O_2_ at rest	None	Floor
III	+	Severe	Fever > 101°F, RR > 25, HR > 110	Multilobar GGO or consolidation	O_2_ > 40%, HFNT, BiPAP	One vasopressor (low level)	ICU
IV	+	Very Severe	Hypotension, SBP < 90 mmHg	Diffuse infiltrates, ARDS	Intubation + PPV; ECMO	≥ 2 vasopressors	ICU

Staging system used to classify COVID-19 severity based on presenting symptoms, radiographic findings, respiratory support requirements, and hemodynamic status. Patients were categorized into five stages (0–IV), ranging from asymptomatic infection (Stage 0) to very severe disease with multiorgan involvement (Stage IV). This framework guided triage decisions, including outpatient management, hospital admission, and ICU level of care.

SX, symptoms; CXR, chest x-ray; HRCT, high-resolution computed tomography; NC, nasal cannula, RR, respiratory rate; bpm, beats per minute; HR, heart rate; SBP, systolic blood pressure; L O_2_, Liters of oxygen; HFNT, high-flow nasal therapy; BiPAP, bilevel positive airway pressure; PPV, positive pressure ventilation; ECMO, extracorporeal membrane oxygenation; GGO, ground-glass opacity; ARDS, acute respiratory distress syndrome.

### 2.3 Data analysis

Statistical analysis was performed using SAS software version 9.4 (SAS Institute Inc., Cary, NC). Continuous variables were summarized using means and standard deviations or medians and interquartile ranges (IQR) as appropriate. These were compared between groups using two-sided t-tests or Wilcoxon rank-sum tests. Categorical variables were expressed as frequencies and percentages and compared using two-sided Chi-square or Fisher’s exact tests as appropriate.

Multivariable logistic regression models were used to assess the association between selected clinical features and the need for ICU care. Results were reported as odds ratios (ORs) with 95% confidence intervals (CIs). Cox proportional hazards models were used to evaluate predictors of mortality during the follow-up period, with results presented as hazard ratios (HRs) and 95% CIs. Kaplan-Meier survival curves were generated to visualize survival probabilities over time, stratified by key BMI categories and hyperglycemia status. Log-rank tests were used to compare survival distributions across groups.

Missing data was handled using complete case analysis; patients with unavailable values for specific variables (e.g., HbA1c) were excluded from those respective analyses. Variables for multivariable models were selected based on clinical relevance and statistical significance in univariate analyses. All statistical tests were two-sided with a p-value of less than 0.05 considered statistically significant.

## 3 Results and discussion

### 3.1 Study population

A total of 516 patients were identified for review based on initial eligibility criteria. 38 patients were excluded after further review; 3 were duplicate encounters, 19 patients only had an emergency room encounter, and the remaining were determined to not have COVID infection upon chart review. 191 (40%) patients were women and 286 patients were men (60%). The mean age was 55.7 years. The majority of patients were Black 38% (n=183), with the remaining 23% Hispanic (n=110), 12% White (n=61), 2.5% (n=12) Asian/Pacific Islander, and 24.5% (n=111) mixed/other race. Body Mass Index (BMI) data was available for 472 patients. Obesity (BMI ≥ 30 kg/m²) was present in 213 patients (45%), while 135 patients (29%) were classified as overweight (BMI 25-29.9 kg/m²).

### 3.2 Mortality data

Of the 478 patients included in the study, 65 (13.6%) were deceased by the end of the follow-up period in August 2022. Deceased patients were significantly older than survivors, with a mean age of 67.9 years compared with 53.8 years among survivors at the end of follow-up (p < 0.001). There was no significant difference in mortality between male and female patients. Racial distribution differed between groups (p = 0.002). Black patients comprised 38.3% of the overall cohort but accounted for 46% of the deceased, while White patients made up 12.8% of the cohort but 24.6% of the deceased, and Hispanic patients represented 26.6% of the cohort but 16.9% of the deceased. Hospital length of stay was significantly longer among deceased patients compared with survivors (14.0 vs. 6.5 days, p = 0.0001), as expected. Deceased patients additionally required greater intensive care utilization, with a mean ICU stay of 106.2 hours versus 20.4 hours among survivors (p = 0.0005). The need for mechanical ventilation was higher in deceased patients (26.2%) compared to survivors (1.5%, p < 0.001). Mean BMI was lower in deceased patients (28.1 kg/m²) compared with survivors (31.0 kg/m²; p = 0.006). Kaplan-Meier analysis demonstrated differences in survival across BMI categories (log-rank p = 0.0002), with lower survival probability observed in the normal weight group compared with overweight and obese groups. (**Figure 1**). A multivariable Cox proportional hazards analysis was performed to evaluate the association between BMI and mortality while accounting for age, sex, race, smoking status, and diabetes diagnosis. BMI was associated with mortality after multivariable adjustment (Wald χ² = 10.24, *p* = 0.006; **Table 2**).

**Figure 1:**
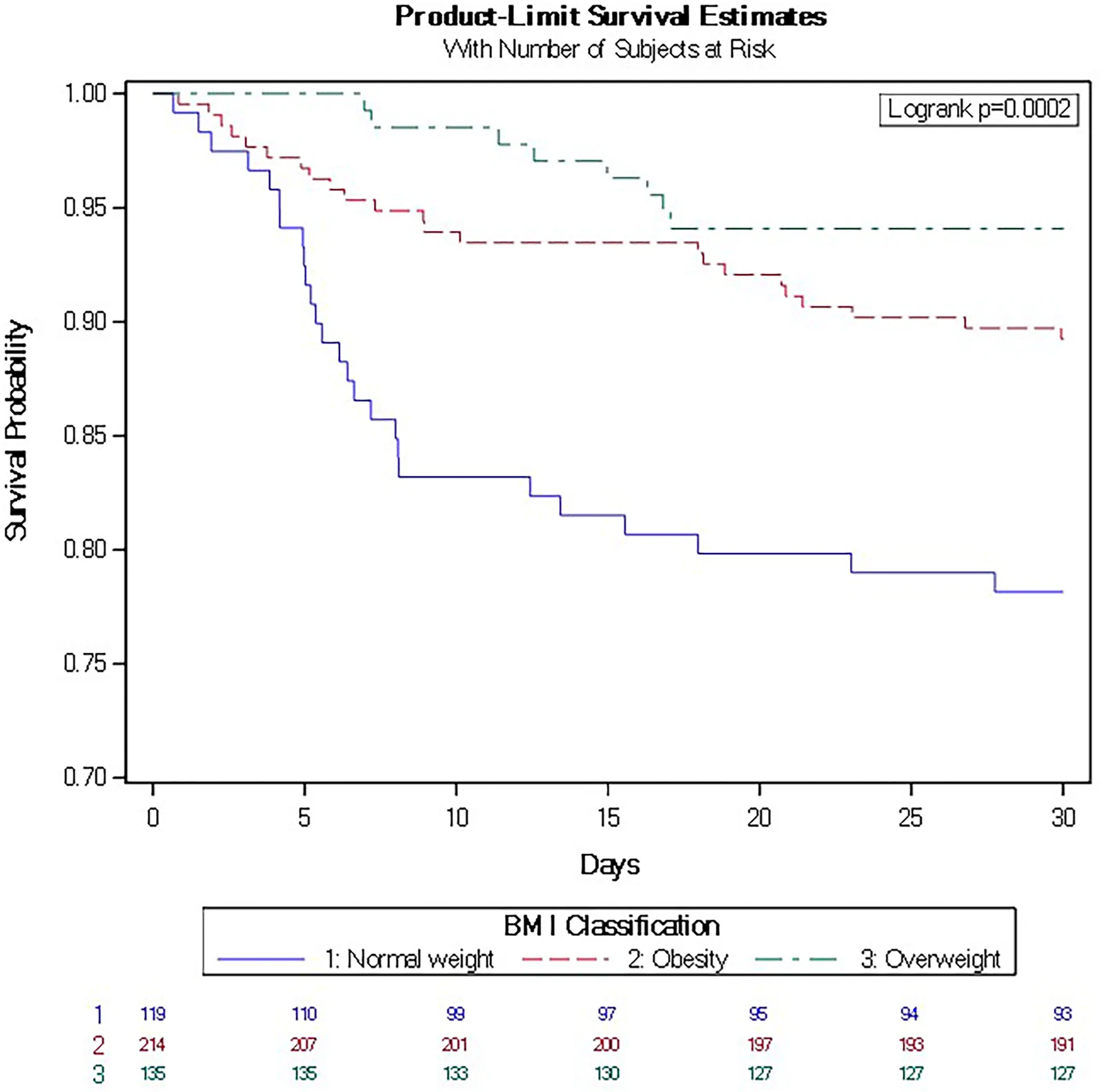
30-day Survival analysis by BMI classification. Kaplan-Meier survival curves stratified by BMI category; normal weight (18–24 kg/m²; blue line), overweight (25-29.9 kg/m²; green line), and obesity (≥30 kg/m²; red line) among hospitalized patients with COVID-19. Survival analysis demonstrated improved survival in overweight and obese patients compared with normal BMI individuals (log-rank p=0.0002).

**Table 2:** Multivariable Cox proportional hazards analysis of mortality according to BMI and clinical characteristics.

Effect	DF	Wald Chi-square	Pr > ChiSq
Age	1	16.7047	< 0.0001
Smoker Status	3	16.0854	0.0011
BMI	2	10.2448	0.0060
Race	4	1.4595	0.8338
Sex	1	0.4931	0.4825
Diabetes Diagnosis	1	1.1842	0.2765

Wald chi-square tests were used to evaluate the association of BMI with mortality after adjustment for age, sex, race, smoking status, and diabetes status. BMI remained significantly associated with mortality after multivariable adjustment (Wald χ² = 10.24, p = 0.006), as did age (Wald χ² = 16.71, p < 0.0001) and smoking status (Wald χ² = 16.09, p = 0.0011). Race, sex, and diabetes status were not statistically significant in the adjusted model. Results are presented as Wald chi-square statistics, degrees of freedom (DF), and corresponding p-values (Pr > ChiSq); probability greater than the chi-square statistic.

There was a significantly higher use of endocrine consults in patients that died (26.2%) compared to those that survived (10.7%) (p = 0.0005). Additionally, there was a higher incidence of diabetes (50.8%) and hypertension (75.4%) in the deceased group compared to the living group (33.9% and 52.5% respectively) (p < 0.001). There was no difference in incidence of new-onset diabetes between the two groups. There was a significantly higher incidence of hyperglycemia in the deceased group (50.8%) compared to the living group (31.2%) (p=0.002). Additionally, there was a much higher incidence of steroid-induced hyperglycemia in the deceased patients (15.4%) compared to the living patients (6.1%, p=0.007). **Figure 2** shows 30-day survival by hyperglycemia status.

**Figure 2:**
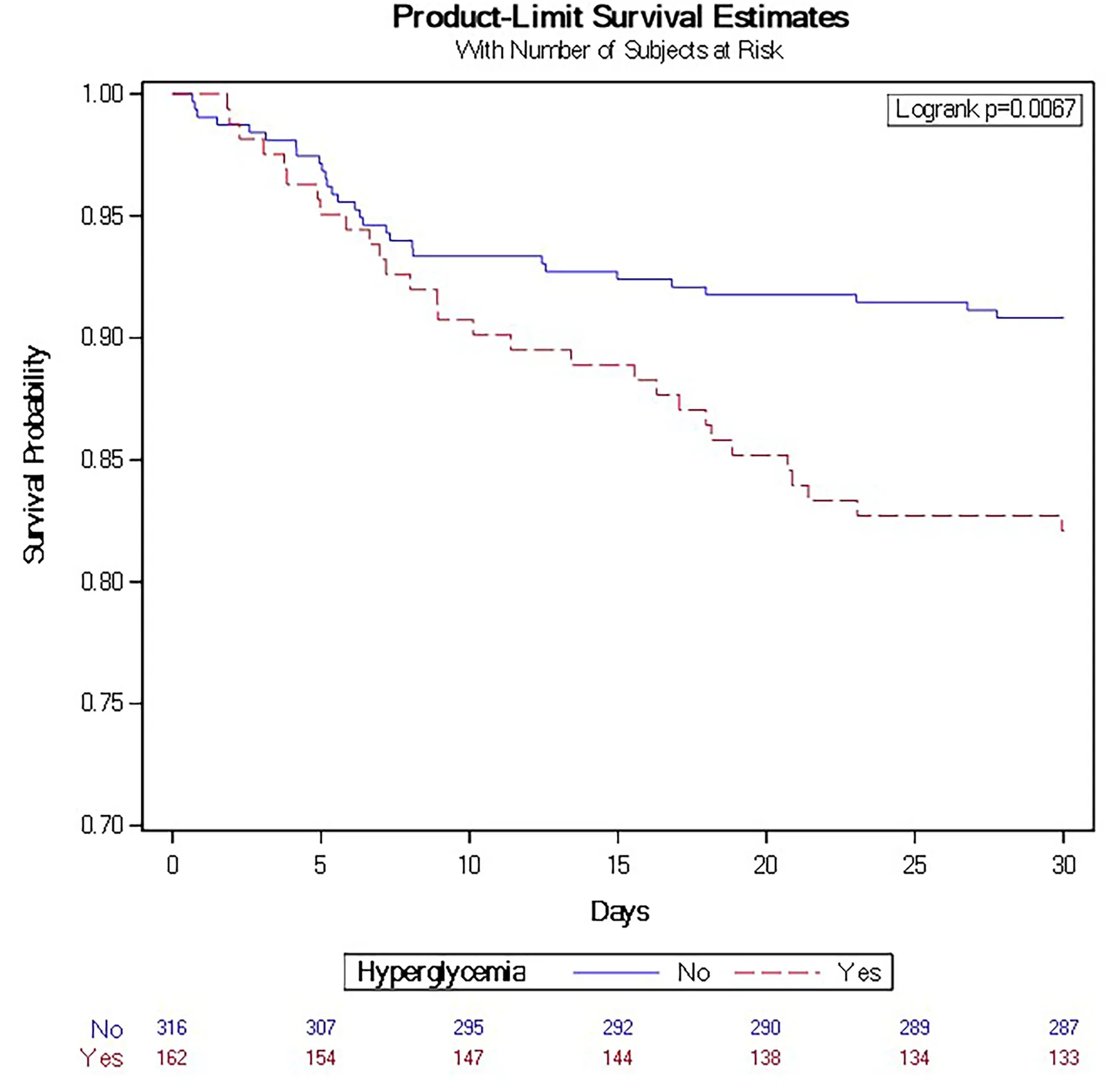
30-day survival analysis by presence of hyperglycemia. Survival curves comparing patients with and without hyperglycemia (as any blood glucose value ≥200 mg/dL (>11.1 mmol/L)) during hospitalization for COVID-19. Survival probability is plotted over a 30-day hospital stay. Patients with hyperglycemia (red dashed line) demonstrated a trend toward lower survival compared to those without hyperglycemia (blue solid line). Log-rank test p = 0.0067.

There was no difference in the mean admission HbA1c among patients who had it performed during admission in the deceased group (n=23, 8%) compared to the living group (n=130, 7.9%) (p=0.86). Similarly, there was no difference in the follow-up HbA1c between the two groups. Mean HbA1c was 7.4% (n=3), 6.6% (n=5), 6.4% (n=3), 6.3% (n=3), 5.7% (n=3) in the deceased patients that had an HbA1c within 3 months of discharge, 3–6 months, 6 to 12 months, 12 to 18 months, 18 months to 24 months respectively compared to 6.8% (n=29), 7.2% (n=29), 7.5% (n=44), 6.9% (n=49), 6.9% (n=53) in the living group respectively. There was a significantly higher use of insulin treatment among deceased patients during admission (63.1%) compared to the living group (37.3%). (p < 0.001). There was no difference in the number of patients treated with steroids in the deceased vs living group however there appeared to be a much higher dosage of methylprednisolone (MP) in the deceased group compared to living group with mean initial daily dose of 100.6 mg in the deceased group compared to 42 mg in the living group. (p = 0.05). There was a similar use of prednisone (% of patients) between the two groups with higher mean initial dose of prednisone in the living group (26.8 mg/day), compared to (18.9 mg/day) in the deceased group (p=0.017). Total mean steroid dose over the admission was significantly higher in the deceased group (1010 mg) compared to the living group (404.8 mg) (p = 0.0001).

Hazard ratios were created through Multiple Cox Regression Analysis amongst hospitalized patients with COVID-19 (**Table 3**).

**Table 3:** Hazard ratios for mortality.

Parameter	Hazard ratio	95% CI	P-value
Weight, kg	0.981	0.969 – 0.994	0.0031
Respiratory rate	1.061	1.028 – 1.096	0.0002
GFR, mL/min/1.73 m²	0.979	0.967 – 0.991	0.0006
Heart Failure	2.266	1.204 – 4.266	0.011
Current Smoker	1.008	0.362 – 2.807	0.99
Former Smoker	2.655	1.406 – 5.013	0.0026

Hazard ratio model identifying predictors of mortality during hospitalization and follow-up. Hazard ratios (HRs) with 95% confidence intervals (CIs) from the multivariable Cox proportional hazards model evaluating predictors of mortality. HR <1 indicates a lower hazard of death, whereas HR >1 indicates a higher hazard of death. Higher respiratory rate and the presence of heart failure or a history of smoking were associated with increased mortality risk. Conversely, higher weight and use of anti-inflammatory agents were associated with a lower hazard of death, suggesting a potential protective effect. Overall, 64 (13.4%) patients were current smokers and 109 (22.8%) were former smokers at the time of admission; smoking status was unknown for 7.1% of patients.

### 3.3 Diabetes mellitus

Out of 478 patients, 173 patients (36%) had preexisting DM. 163 patients had type 2 diabetes, 4 had type 1 diabetes and the remaining had an unknown type of diabetes. 27 patients (5.6%) had prediabetes. 20 patients had new-onset diabetes determined from chart review. Patients with diabetes were significantly older (mean age 61.8 years) compared to those without diabetes (52.3 years) (p < 0.001). A significantly larger number of patients with DM required mechanical ventilation compared with those without diabetes (8.7% vs 2.6%, p < 0.001). 6.1% of patients were readmitted for COVID-19 within 90 days of discharge. Of those, 6.4% had diabetes diagnosis compared to 5.9% without diabetes (p = 0.84). 127 (73.4%) of patients with diabetes had hyperglycemia, compared to 35 (11.5%) of patients without diabetes. (p < 0.001). There was an increased use of immunosuppressants in patients with diabetes, n= 48 (27.7%) compared to patients without diabetes, n=60 (19.7%) (p = 0.043). The initial mean daily steroid dose was the same in patients with and without diabetes at 40 mg/day of methylprednisolone equivalents. Mean steroid use over the entire admission was 569.7 mg of methylprednisolone in patients with diabetes compared to 438.4 mg in patients without diabetes (p = 0.085). Our study confirmed the observations from other studies that patients with diabetes had more severe disease as evidenced by statistically significant increased use of mechanical ventilation and increased use of immunosuppressants.

### 3.4 HbA1c data

A majority of patients did not have a HbA1c test done during the admission. 153 patients (32%) had HbA1c on admission of which 27.2% of patients with diabetes had HbA1c > 8%. Median HbA1c was 7.8% among patients with diabetes compared to 6.3% among those without diabetes (p <0.001). Among patients that had an HbA1c ≥ 6.5% (n=100), median HbA1c was 8.25%. 148 patients had an HbA1c done prior to admission. Median preadmission HbA1c was 7.2% in patients with diabetes compared to 5.7% in those without diabetes (p < 0.001). 32 patients (11 with diabetes) had an HbA1c within 3 months of discharge, 34 patients (19 with diabetes) between 3–6 months, 46 patients (26 with diabetes) between 6 to 12 months, 52 patients (29 with diabetes) between 12 to 18 months, and 54 patients (28 with diabetes) between 18 months to 24 months. **Table 4** depicts median follow up HbA1c levels over time.

**Table 4:** Median follow-up HbA1c by diabetes status and time since discharge.

Diabetes status	≤ 3 months	3–6 months	6–12 months	12–18 months	18–24 months
Diabetes	7.4% (n=11)	7.2% (n=19)	7.7% (n=26)	7.0% (n=29)	7.2% (n=28)
No Diabetes	5.8% (n=21)	5.9% (n=15)	5.9% (n=20)	5.7% (n=23)	5.7% (n=26)

HbA1c values at ≤ 3, 3-6, 6-12, 12-18, and 18–24 months following discharge are shown stratified by diabetes status.

### 3.5 ICU data

93 of 478 patients required ICU care during the hospitalization. Mean age of patients requiring ICU care was higher at 58.9 years compared to 54.9 years in those that did not require ICU care (p = 0.028). There was a similar percentage of male and female patients requiring ICU care and no difference in race distribution. Mean BMI was similar in patients requiring ICU care (30.5 kg/m²) compared to those that did not require ICU care (31.1 kg/m²). Mean weight was similar between the groups as well. There was an increased use of endocrine and rheumatology consults in patients needing ICU care (37.6% vs 6.8%) and (48.4 vs 10.6%) respectively. This is likely related to more severe illness and higher incidence of cytokine storm in the critically ill (p <0.001). There was additionally a higher percentage of patients with diabetes (46.2%) among those admitted to ICU compared to those that were not (34%) (p = 0.045). There was also a significantly higher incidence of hyperglycemia and steroid-induced hyperglycemia in patients requiring ICU care (61.3% and 21.5%) vs. no ICU care (26.5% vs 3.9%; p < 0.0001). There was a higher incidence of hypertension 65.6% in the ICU group compared to 53.2% in the non-ICU group (p = 0.032). There was also a much higher incidence of sleep apnea in the ICU group (15.1%) compared to the non-ICU group (3.4%). There was no significant difference in the Hba1c during admission or follow-up in the ICU and non-ICU groups. There was a significantly higher mean dose of steroids used during the admission in patients who required ICU (1401.5 mg of MP) compared to patients who did not require ICU care (264.3mg MP) (p < 0.0001).

Multiple logistic regression models were created to determine Odds Ratios for needing ICU care (**Figure 3**). Of note, Odds Ratio for a diagnosis of sleep apnea was 4.346 and for steroid-induced hyperglycemia was 3.532 indicating that this diagnosis appeared to correlate with requiring greater need for ICU care. Sleep apnea appeared to be a risk factor for more severe illness in other studies as well (4).

**Figure 3:**
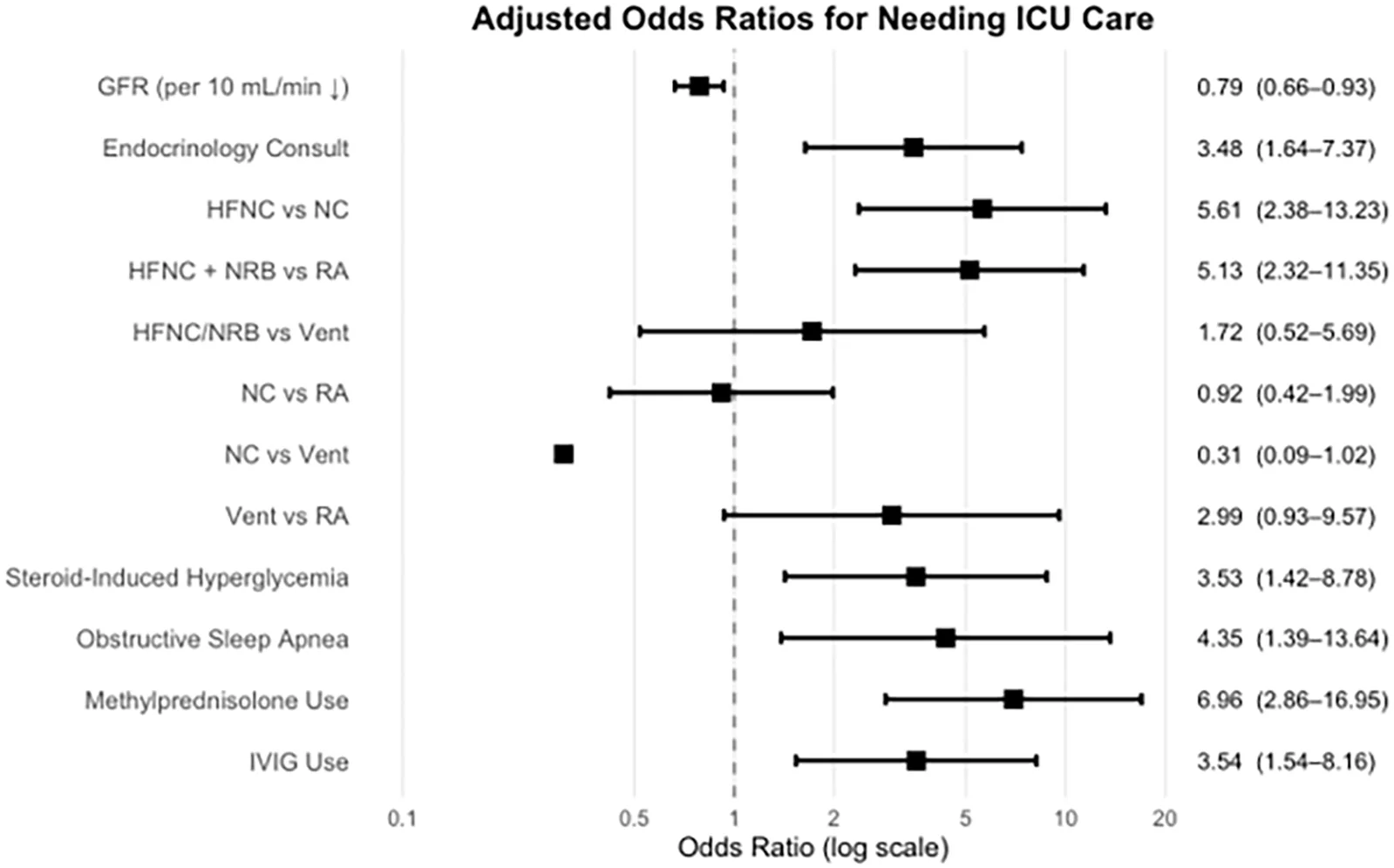
Predictors of ICU admission in hospitalized patients with COVID-19. Multivariable model assessing predictors of ICU care. Significant associations included endocrinology consultation (OR 3.48, 95% CI 1.64-7.37), steroid-induced hyperglycemia (OR 3.53, 95% CI 1.42-8.78), obstructive sleep apnea (OR 4.35, 95% CI 1.39-13.64), methylprednisolone use (OR 6.96, 95% CI 2.86-16.95), and IVIG use (OR 3.54, 95% CI 1.54-8.16). Decreasing GFR was also associated with increased ICU risk (per 10 mL/min ↓, OR 0.79, 95% CI 0.66-0.93).

### 3.6 Cytokine storm

67 patients (14%) had cytokine storm which was identified by rheumatology consult using clinical conditions, inflammatory marker values or the use of immunosuppressants; sarilumab, tocilizumab or anakinra. 30.8% of patients with cytokine storm were deceased at the end of follow-up compared to 11.4% of those that survived (p < 0.001), indicating more severe disease as expected.

### 3.7 Endocrinology consult

61 patients (12.8%) had an endocrinology consult. A larger number of patients with diabetes (22%) had endocrine consults compared to 15.7% of those without diabetes as expected (p < 0.001).

## 4 Discussion

Our study highlights several key points in an urban center of hospitalized patients with diabetes mellitus and COVID-19 infection during the worldwide pandemic. Approximately one-third of our study patients had pre-existing diabetes, with the majority of those being type 2 DM, while only a small percentage had new onset DM determined by chart review. Patients with diabetes were significantly older, with a mean admission HbA1c of 7.8% among those who had levels drawn. A small percentage of patients required hospital readmission within 3 months of discharge, with a higher incidence of readmission among those with diabetes, however, this difference was not statistically significant and is likely due to limited sample size. This study confirms prior observations that patients with diabetes hospitalized with COVID-19 are more likely to develop severe disease, as evidenced by increased requirements for ICU care, mechanical ventilation, and increased use of immunosuppressants. These findings are consistent with previous reports demonstrating that obesity may exacerbate infection severity in acute illnesses such as sepsis and community-acquired pneumonia (5, 6).

The relationship between baseline HbA1c and COVID-19 severity and mortality remains debated. Prior studies have demonstrated mixed findings regarding the prognostic value of HbA1c in this setting. Min et al. performed a retrospective cohort study of 168,803 patients with diabetes stratified by HbA1c, demonstrating an increased risk of COVID-19 hospitalization with higher HbA1c, but no consistent increase in mortality (7). Another study has suggested that admission random glucose may be a better predictor of mortality than HbA1c (8). Together, these findings highlight the complexity of glycemic markers in predicting outcomes in patients with COVID-19. Although we initially aimed to evaluate the impact of COVID-19 hospitalization on follow-up HbA1c levels, this analysis was not feasible due to the small sample size, as HbA1c measurements were not routinely drawn on admission. These findings support the need to standardize HbA1c measurement as part of inpatient admission protocols.

In our cohort, we observed a higher incidence of diabetes among deceased patients compared to the surviving population, conferring a higher mortality rate in this group. Deceased patients were also significantly older and more likely to be of Black race, with no significant difference in mortality between male and female sex. There was no difference in the number of patients treated with steroids in the deceased vs the living group. Notably, our study is particularly unique as each patient in our institution received steroids as part of a hospital-wide COVID-19 treatment protocol, with dosing based on disease severity. This likely explains the higher mean initial daily dose of methylprednisolone in the deceased group compared to the living group (100.6 mg vs. 42 mg, p = 0.05), reflecting greater underlying disease severity rather than an independent effect of corticosteroid exposure. Similarly, the significantly higher cumulative steroid dose observed among patients who died and those requiring ICU care should be interpreted in the context of confounding by indication, as patients with more severe COVID-19 were more likely to receive higher-intensity corticosteroid therapy. Higher steroid exposure may also have accounted for the increased incidence of hyperglycemia and steroid-induced hyperglycemia observed among critically ill patients; however, these associations cannot be interpreted as causal given the strong relationship between corticosteroid exposure and disease severity. Because corticosteroids were incorporated into the institutional treatment protocol, an untreated comparison group was not available to assess the independent effect of corticosteroid therapy.

Interestingly, our study showed higher BMI; > 25 kg/m² (overweight and obese categories) was associated with improved survival compared with normal BMI (**Figure 4**). Additionally, our 30-day survival analysis demonstrated improved survival among both overweight and obese patients compared with normal-weight individuals. These findings are similar to a population-based cohort study from England that examined the association between BMI and COVID-19 mortality among patients with type 1 and type 2 diabetes mellitus (9). The association between BMI and COVID-19 mortality was U-shaped for both type 1 and type 2 diabetes mellitus, with the lowest risk observed among patients with a BMI of 25.0-29.9 kg/m². In contrast, Cariou et al. reported in a nationwide, pre-COVID-19 vaccination study conducted at French centers that increasing BMI was associated with an increased risk of tracheal intubation or death within 7 days of hospital admission (4). However, the patient population and clinical setting were very different and may reflect differences in demographic, socioeconomic, and clinical management factors in our urban population. Similarly, in a community-based cohort study in England, Altunkaya et al. reported longer hospital stays and higher likelihood of being admitted to ICU in patients with obesity (BMI ≥ 30 kg/m²) in a predominantly unvaccinated COVID-19 population, with approximately 81% of patients unvaccinated (10). The findings from our study should also be considered in the context of the broader pre-vaccine COVID-19 literature examining the relationship between BMI, obesity, and clinical outcomes. Several early observational studies reported an association between obesity and increased disease severity, ICU admission, mechanical ventilation, and mortality, particularly among hospitalized patients, however, subsequent studies have reported differing findings, including potential survival advantages among patients with obesity in selected populations. A summary of selected studies examining BMI and obesity in relation to COVID-19 outcomes is presented in **Table 5**.

**Figure 4:**
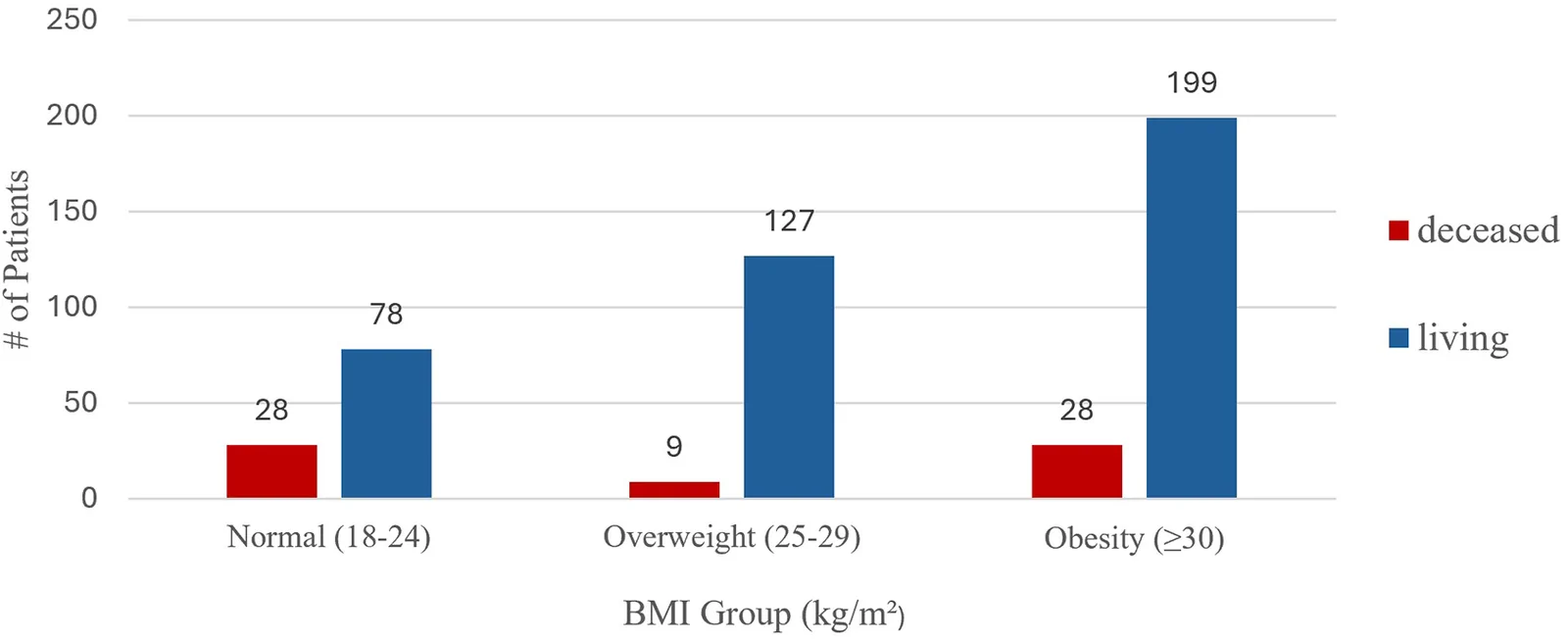
Mortality distribution across BMI Categories. Comparison of living and deceased patients grouped by BMI class (normal weight: 18-24.9 kg/m², overweight: 25-29.9 kg/m², obesity: >30 kg/m²). Higher BMI categories showed fewer deaths, consistent with an observed obesity paradox.

**Table 5:** Selected pre-vaccine studies examining BMI/obesity and COVID-19 outcomes.

Study	Setting/period	Sample	Findings
Simonnet et al., 2020 (15)	Lille, France; March 2020	124 ICU patients	Obesity was highly prevalent among patients admitted to the ICU. The proportion requiring invasive mechanical ventilation increased with BMI, with the highest frequency among patients with BMI >35 kg/m². BMI was independently associated with invasive mechanical ventilation after adjustment for age, diabetes, and hypertension.
Petrilli et al., 2020 (16)	New York City; March-April 2020	5,279 patients	In a large New York City cohort, BMI and obesity-related factors were associated with hospitalization and progression to critical illness.
Palaiodimos et al., 2020 (17)	Bronx, New York; March-April 2020	Hospitalized COVID-19 patients	Severe obesity was independently associated with worse in-hospital outcomes and higher mortality in a predominantly urban, ethnically diverse population.
Cariou et al., 2020 (4)	France; March-April 2020	1,965 patients with diabetes	Among hospitalized patients with diabetes, obesity and other clinical characteristics were associated with adverse early COVID-19 outcomes.
Holman et al., 2020 (9)	England; Jan 2017 - May 2020	Population-based cohort of people with type 1 (264,390) and type 2 diabetes (2,874,020)	Large population-based analysis identified clinical and demographic factors associated with COVID-19-related mortality among people with diabetes, supporting the increased risk associated with diabetes while also evaluating obesity and other potentially modifiable risk factors.
Du et al., 2021 (18)	Multiple observational cohorts	109,881 patients from 16 studies	Dose-response meta-analysis demonstrated an association between higher BMI and critical COVID-19 and in-hospital mortality, supporting obesity as an important risk factor for adverse outcomes.
Sprockel Díaz et al., 2023 (14)	Colombia; prospective cohorts	402 hospitalized patients; 302 ICU patients	Obesity was more common among patients admitted to the ICU. Among ICU patients, mortality was lower among patients with obesity, although obesity was not independently associated with ICU admission or mortality after multivariable adjustment.

Summary of selected observational studies, meta-analyses, and review literature evaluating the relationships between BMI and obesity and COVID-19 severity, intensive care utilization, mechanical ventilation, and mortality.

Our findings could be specifically related to the underserved population of North Philadelphia and can potentially be a result of the obesity paradox. The obesity paradox is defined as an association where in which overweight or obese (defined by BMI) is linked to higher survival rates compared to patients with normal BMI or underweight BMI categories. Although elevated BMI is typically a risk factor for many diseases, it is thought to be protective for older patients with certain conditions such as myocardial infarction, stroke, heart failure and even cancer although these specific comorbidities were not examined in our study population. There have been some studies supporting this in the urban populations specifically. One study of patients admitted to an urban medical center with community-acquired infectious diseases found that overweight and obese patients had significantly lower 90-day and long-term (one-year and five-year) mortality rates compared to normal weight individuals (11). An observational cohort study looked at older obese Medicare beneficiaries with severe sepsis and showed survival benefits in obese patients at one year compared to patients of normal weight (12). Proposed mechanisms of this paradox include greater metabolic reserves (large energy stores), potential protective adipokines (adiponectin may have cardioprotective effects), and increased muscle mass (improved cardiorespiratory fitness) (12).

On the contrary, the obesity paradox is increasingly understood as a result of statistical biases rather than a beneficial effect of higher BMI in individuals with diabetes. One proposed explanation is survival or selection bias, in which healthier individuals with overweight or obesity are more likely to survive to older age or to the development of chronic disease, thereby being overrepresented among hospitalized cohorts. In addition, patients with overweight or obesity may receive more aggressive clinical management, particularly with respect to respiratory support, which could influence observed outcomes. Patients with COVID and overweight or obesity may have been treated differently at our institution. They were more likely to have respiratory distress, more commonly placed on high flow oxygen, BiPAP or placed in the prone position and had a lower threshold to receive ICU or step down level of care. It is important to note that BMI is an imperfect measure and does not differentiate between fat mass and lean mass. Patients with severe illnesses may experience unintentional weight loss, leading to a lower BMI further complicating this paradox.

Overall, the existence and extent of the obesity paradox remains a subject of ongoing debate, particularly in patients with obesity, diabetes, and COVID-19 infection. While some studies suggest potential survival benefits of higher BMI in certain populations, these findings should be interpreted in the context of the well-established long-term risks of obesity (13). To our knowledge, this is the first study examining the obesity paradox in an urban population with both diabetes and COVID-19 infection.

Strengths of this study include the use of two independent reviewers, a unique and underserved population, detailed clinical data capturing demographics characteristics, comorbidities, and the use of a standardized hospital-wide steroid treatment protocol for all patients, minimizing treatment variability. Limitations include its retrospective design, a relatively short study duration, modest sample size, and potential intervention bias, particularly in the analysis of BMI and survival. Patients with overweight or obesity may have been perceived to be at higher risk for respiratory deterioration and may therefore have received more intensive pulmonary interventions. These differences in clinical management may have influenced outcomes and could contribute to the observed association between higher BMI and improved survival. Although the standardized steroid protocol reduced treatment variability, all patients received corticosteroids, and steroid dosing was influenced by disease severity. Therefore, the higher cumulative steroid doses observed among patients who died or required ICU care may reflect greater disease severity rather than a direct effect of corticosteroid exposure. Additionally, most patients did not have a HbA1c measured during or after the admission, limiting assessment of glycemic trends. Finally, being a single-center study with a modest sample size for some analyses also limits the generalizability of the findings.

## 5 Conclusion

In conclusion, among patients hospitalized with COVID-19 in an urban center, diabetes, older age, Black race, and need for ICU-level care were more likely to have higher mortality. Paradoxically, higher BMI was associated with improved survival in this population. Close monitoring of patients with diabetes or hyperglycemia during COVID-19 hospitalization is warranted due to their increased risk of adverse outcomes. Future research should further explore the long-term effects of COVID-19 on glycemia and the complex interplay between COVID-19, diabetes mellitus, and obesity.

Wald chi-square tests were used to evaluate the association of BMI with mortality after adjustment for age, sex, race, smoking status, and diabetes status. BMI remained significantly associated with mortality after multivariable adjustment (Wald χ² = 10.24, p = 0.006), as did age (Wald χ² = 16.71, p < 0.0001) and smoking status (Wald χ² = 16.09, p = 0.0011). Race, sex, and diabetes status were not statistically significant in the adjusted model. Results are presented as Wald chi-square statistics, degrees of freedom (DF), and corresponding p-values (Pr > ChiSq); probability greater than the chi-square statistic.

## Data Availability

The raw data supporting the conclusions of this article will be made available by the authors, without undue reservation.
