# Supplementary material for: Body mass index, diabetes, inflammation and survival in COVID-19: a retrospective study evaluating the obesity paradox in an urban hospital center
Source: Front Clin Diabetes Healthc. 2026 Oct 13;7:1946918. doi: 10.3389/fcdhc.2026.1946918 (PMC13710009; doi:10.3389/fcdhc.2026.1946918)
Supplement: Supplementary file 1 [file Table1.docx]

**Supplementary Tables**

**Supplementary Table 1: Patient Demographics and Characteristics Stratified by Diabetes Diagnosis**

Baseline demographic and baseline clinical characteristics of hospitalized patients with COVID-19 (n=478), stratified by presence or absence of diabetes mellitus. Continuous variables are reported as mean (SD) and categorical variables as N (%), with comparisons performed using t-test or chi-square test.

| **Variable** | **Missing Value** | **Overall**  **(n=478)** | **Yes**  **(n=173)** | **No**  **(n=305)** | **Chi-Square or T-test**  **p-value** |
| --- | --- | --- | --- | --- | --- |
| Age (at admission), N | 0 | 478 | 173 | 305 | **<.0001** |
| Mean (SD) |  | 55.7 (15.7) | 61.8 (12.9) | 52.3 (16.1) |  |
| Sex, N (%) | 0 |  |  |  | **0.021** |
| Female |  | 191 (40.0%) | 81 (46.8%) | 110 (36.1%) |  |
| Male |  | 287 (60.0%) | 92 (53.2%) | 195 (63.9%) |  |
| Race, N (%) | 0 |  |  |  | 0.21 |
| Black |  | 183 (38.3%) | 78 (45.1%) | 105 (34.4%) |  |
| Hispanic |  | 127 (26.6%) | 43 (24.9%) | 84 (27.5%) |  |
| Other |  | 107(22.4%) | 32(18.5%) | 75 (24.6%) |  |
| White |  | 61 (12.8%) | 20 (11.6%) | 41 (13.4%) |  |
|  |  |  |  |  |  |
| BMI, N | 6 | 472 | 172 | 300 |  |
| Mean (SD) |  | 30.6 (8.1) | 31.3 (8.1) | 30.2 (8.0) | 0.15 |
| Obesity, N (%) | 0 |  |  |  | 0.15 |
| N |  | 264 (55.2%) | 88 (50.9%) | 176 (57.7%) |  |
| Y |  | 214 (44.8%) | 85 (49.1%) | 129 (42.3%) |  |
| Weight, N | 5 | 473 | 173 | 300 | 0.63 |
| Mean (SD) |  | 89.5 (45.7) | 88.3 (24.9) | 90.1 (54.2) |  |

**Supplementary Table 2: Diabetes-Related Characteristics Stratified by Diabetes Diagnosis**
Admission glycemic indices, diabetes subtype, diabetes therapies, endocrine consultation rates, longitudinal HbA1c trends, and inpatient steroid exposure among patients with and without diabetes. Continuous variables are expressed as mean (SD) and categorical variables as N (%).

| **Variable** | **Missing Value** | **Overall**  (n=478) | **Yes**  (n=173) | **No**  (n=305) | **Chi-Square or T-test**  p-value |
| --- | --- | --- | --- | --- | --- |
| T2DM, N (%) | 1 |  |  |  |  |
| N |  | 303 (63.5%) | 9 (5.2%) | 294 (96.4%) | **<.0001** |
| Y |  | 174 (36.5%) | 163 (94.8%) | 11 (3.6%) |  |
| Prediabetes, N (%) | 0 |  |  |  |  |
| N |  | 451 (94.4%) | 172 (99.4%) | 279 (91.5%) | **0.0003** |
| Y |  | 27 (5.6%) | 1 (0.6%) | 26 (8.5%) |  |
| Admission A1C, N (%) | 0 |  |  |  | **<.0001** |
| 6.5-7.9 |  | 49 (10.3%) | 44 (25.4%) | 5 (1.6%) |  |
| <=6.4 |  | 52 (10.9%) | 11 (6.4%) | 41 (13.4%) |  |
| >=8.0 |  | 52 (10.9%) | 47 (27.2%) | 5 (1.6%) |  |
| Not Collected |  | 325 (68.0%) | 71 (41.0%) | 254 (83.3%) |  |
| Glucose, N (% | 0 |  |  |  | **<.0001** |
| 200-300 mg/dL  11.1-16.7 mmol/L |  | 49 (8.8%) | 28 (16.2%) | 14 (4.6%) |  |
| <200 mg/dL  <11.1 mmol/L |  | 403 (84.3%) | 114 (65.9%) | 289 (94.8%) |  |
| >300 mg/dL  >16.7 mmol/L |  | 33 (6.9%) | 31 (17.9%) | 2 (0.7%) |  |
| Metformin, N (%) | 0 |  |  |  | **<.0001** |
| N |  | 431 (90.2%) | 130 (75.1%) | 301 (98.7%) |  |
| Y |  | 47 (9.8%) | 43 (24.9%) | 4 (1.3%) |  |
| Insulin, N (%) | 0 |  |  |  | **<.0001** |
| N |  | 283 (59.2%) | 25 (14.5%) | 258 (84.6%) |  |
| Y |  | 195 (40.8%) | 148 (85.5%) | 47 (15.4%) |  |
| Endocrine consult, N (%) | 0 |  |  |  | **<.0001** |
| N |  | 417 (87.2%) | 122 (70.5%) | 295 (96.7%) |  |
| Y |  | 61 (12.8%) | 51 (29.5%) | 10 (3.3%) |  |
| Post-discharge  HbA1c |  |  |  |  |  |
| HbA1c 3m, N (%) | 446 | 32 | 18 | 14 | **0.082** |
| Mean (SD) |  | 6.9 (1.7) | 7.3 (1.7) | 6.3 (1.6) |  |
| HbA1c 3-6m, N (%) | 444 | 34 | 19 | 15 | **0.003** |
| Mean (SD) |  | 7.1 (1.8) | 7.8 (1.8) | 6.1 (1.2) |  |
| HbA1c 6-12m, N (%) | 431 | 47 | 26 | 21 | **0.002** |
| Mean (SD) |  | 7.5 (2.3) | 8.4 (2.4) | 6.4 (1.7) |  |
| HbA1c 12-18m, N (%) | 426 | 52 | 29 | 23 | **<.0001** |
| Mean (SD) |  | 6.9 (1.9) | 7.8 (2.2) | 5.8 (0.6) |  |
| HbA1c >18m, N (%) | 424 | 54 | 28 | 26 | **0.0001** |
| Previous HbA1c, N (%) | 330 | 148 | 78 | 70 | **<.0001** |
| Mean (SD) |  | 7.1 (2.5) | 8.3 (2.8) | 5.7 (0.8) |  |
| Admission  HbA1c, N (%) | 325 | 153 | 102 | 51 |  |
| Mean (SD) |  | 7.9 (2.4) | 8.7 (2.5) | 6.3 (1.3) | **<.0001** |
| Steroids Received, N (%) | 0 |  |  |  |  |
| N |  | 129 (27.0%) | 42 (24.3%) | 87 (28.5%) | 0.31 |
| Y |  | 349 (73.0%) | 131 (75.7%) | 218 (71.5%) |  |
| Initial total daily steroid dose, N (%) | 0 | 478 | 173 | 305 |  |
| Mean (SD) |  | 49.3 (87.3) | 48.2 (85.2) | 50.0 (88.5) | 0.83 |

**Supplementary Table 3: Patient Comorbidities Stratified by Diabetes Diagnosis**
Pre-existing comorbidities, inflammatory biomarkers, respiratory support requirements, and readmission outcomes among hospitalized patients with COVID-19, stratified by diabetes status. Statistical comparisons were performed using chi-square tests for categorical variables and t-tests for continuous variables.

| **Variable** | **Missing Value** | **Overall**  **(n=478)** | **Yes**  **(n=173)** | **No**  **(n=305)** | **Chi-Square or T-test**  **p-value** |
| --- | --- | --- | --- | --- | --- |
| Microvascular complications, N (%) | 0 |  |  |  |  |
| N |  | 424 (88.7%) | 123 (71.1%) | 301 (98.7%) | **<.0001** |
| Y |  | 54 (11.3%) | 50 (28.9%) | 4 (1.3%) |  |
| Macrovascular complications, N (%) | 0 |  |  |  |  |
| N |  | 435 (91.0%) | 132 (76.3%) | 303 (99.3%) | **<.0001** |
| Y |  | 43 (9.0%) | 41 (23.7%) | 2 (0.7%) |  |
| Hyperglycemia, N (%) | 0 |  |  |  |  |
| No |  | 316 (66.1%) | 46 (26.6%) | 270 (88.5%) | **<.0001** |
| Yes |  | 162 (33.9%) | 127 (73.4%) | 35 (11.5%) |  |
| Steroid induced hyperglycemia, N (%) | 0 |  |  |  |  |
| N |  | 443 (92.7%) | 171 (98.8%) | 272 (89.2%) | **<.0001** |
| Y |  | 35 (7.3%) | 2 (1.2%) | 33 (10.8%) |  |
| Dyslipidaemia, N (%) | 0 |  |  |  |  |
| N |  | 338 (70.7%) | 90 (52.0%) | 248 (81.3%) | **<.0001** |
| Y |  | 140 (29.3%) | 83 (48.0%) | 57 (18.7%) |  |
| Interstitial Lung disease, N (%) | 0 |  |  |  |  |
| N |  | 434 (90.8%) | 154 (89.0%) | 280 (91.8%) | 0.31 |
| Y |  | 44 (9.2%) | 19 (11.0%) | 25 (8.2%) |  |
| COPD, N (%) | 0 |  |  |  |  |
| N |  | 425 (88.9%) | 154 (89.0%) | 271 (88.9%) | 0.96 |
| Y |  | 53 (11.1%) | 19 (11.0%) | 34 (11.1%) |  |
| HTN, N (%) | 0 |  |  |  |  |
| N |  | 212 (44.4%) | 42 (24.3%) | 170 (55.7%) | **<.0001** |
| Y |  | 266 (55.6%) | 131 (75.7%) | 135 (44.3%) |  |
| HF, N (%) | 0 |  |  |  |  |
| N |  | 412 (86.2%) | 141 (81.5%) | 271 (88.9%) | **0.025** |
| Y |  | 66 (13.8%) | 32 (18.5%) | 34 (11.1%) |  |
| Cancer History, N (%) | 0 |  |  |  |  |
| N |  | 447 (93.5%) | 161 (93.1%) | 286 (93.8%) | 0.76 |
| Y |  | 31 (6.5%) | 12 (6.9%) | 19 (6.2%) |  |
| CKD, N (%) | 0 |  |  |  |  |
| N |  | 386 (80.8%) | 125 (72.3%) | 261 (85.6%) | **0.0004** |
| Y |  | 92 (19.2%) | 48 (27.7%) | 44 (14.4%) |  |
| ERSD on Dialysis, N (%) | 0 |  |  |  |  |
| N |  | 446 (93.3%) | 158 (91.3%) | 288 (94.4%) | 0.19 |
| Y |  | 32 (6.7%) | 15 (8.7%) | 17 (5.6%) |  |
| Black, eGFR, N (%) | 11 | 467 | 170 | 297 |  |
| Mean (SD) |  | 53.1 (19.6) | 52.0 (25.2) | 53.7 (15.5) | 0.43 |
| Non-Black_eGFR, N (%) | 12 | 466 | 169 | 297 |  |
| Mean (SD) |  | 50.9 (17.1) | 48.4 (18.2) | 52.3 (16.2) | **0.015** |
| Asthma, N (%) | 0 |  |  |  |  |
| N |  | 438 (91.6%) | 158 (91.3%) | 280 (91.8%) | 0.86 |
| Y |  | 40 (8.4%) | 15 (8.7%) | 25 (8.2%) |  |
| Sleep Apnea, N (%) | 0 |  |  |  |  |
| N |  | 451 (94.4%) | 160 (92.5%) | 291 (95.4%) | 0.18 |
| Y |  | 27 (5.6%) | 13 (7.5%) | 14 (4.6%) |  |
| Smoker Status, N (%) | 0 |  |  |  |  |
| Current Smoker |  | 64 (13.4%) | 14 (8.1%) | 50 (16.4%) | **0.016** |
| Former Smoker |  | 109 (22.8%) | 50 (28.9%) | 59 (19.3%) |  |
| Never Smoker |  | 271 (56.7%) | 98 (56.6%) | 173 (56.7%) |  |
| Unknown |  | 34 (7.1%) | 11 (6.4%) | 23 (7.5%) |  |
| Rheum Consult, N (%) | 0 |  |  |  | **0.088** |
| N |  | 392 (82.0%) | 135 (78.0%) | 257 (84.3%) |  |
| Y |  | 86 (18.0%) | 38 (22.0%) | 48 (15.7%) |  |
| Cytokine Storm, N (%) | 0 |  |  |  | 0.19 |
| N |  | 411 (86.0%) | 144 (83.2%) | 267 (87.5%) |  |
| Y |  | 67 (14.0%) | 29 (16.8%) | 38 (12.5%) |  |
| CRP, N (%) | 50 | 428 | 151 | 277 | 0.59 |
| Mean (SD) |  | 13.0 (27.6) | 14.0 (26.3) | 12.5 (28.3) |  |
| D-Dimer, N (%) | 32 | 446 | 159 | 287 | 0.90 |
| Mean (SD) |  | 2.9 (10.0) | 2.9 (8.1) | 3.0 (10.9) |  |
| Ferritin, N (%) | 59 | 419 | 154 | 265 | 0.24 |
| Mean (SD) |  | 844.0 (2353.3) | 704.6 (724.0) | 925.1 (2906.4) |  |
| IL6, N | 256 | 222 | 86 | 136 |  |
| Mean (SD) |  | 267.2 (2829.8) | 132.0 (464.8) | 352.8 (3599.1) | 0.48 |
| Creatinine, N (%) | 14 | 464 | 171 | 293 | 0.16 |
| Mean (SD) |  | 5.0 (38.9) | 2.5 (5.6) | 6.5 (48.7) |  |
| Readmit for COVID, N (%) | 0 |  |  |  |  |
| N |  | 449 (93.9%) | 162 (93.6%) | 287 (94.1%) | 0.84 |
| Y |  | 29 (6.1%) | 11 (6.4%) | 18 (5.9%) |  |
| Readmit for Non-COVID, N (%) | 0 |  |  |  |  |
| N |  | 369 (77.2%) | 132 (76.3%) | 237 (77.7%) | 0.73 |
| Y |  | 109 (22.8%) | 41 (23.7%) | 68 (22.3%) |  |
| O2 Intervention, N (%) | 0 |  |  |  |  |
| HFNC/NRB |  | 60 (12.6%) | 21 (12.1%) | 39 (12.8%) | **0.001** |
| NC |  | 111 (23.2%) | 50 (28.9%) | 61 (20.0%) |  |
| RA |  | 284 (59.4%) | 87 (50.3%) | 197 (64.6%) |  |
| VENT |  | 23 (4.8%) | 15 (8.7%) | 8 (2.6%) |  |

**Supplemental Table 7: Patient’s COVID-19 Confirmation Stratified by Diabetes Diagnosis**

SARS-CoV-2 PCR confirmation rates and chest CT severity scores among hospitalized patients, stratified by diabetes status. Values are presented as N (%) with chi-square testing used for between-group comparisons.

| **Variable** | **Missing Value** | **Overall**  (n=478) | **Yes**  (n=173) | **No**  (n=305) | **Chi-Square or T-test**  p-value |
| --- | --- | --- | --- | --- | --- |
| COVID SWAB POSITIVE , N (%) | 0 |  |  |  |  |
| N |  | 58 (12.1%) | 18 (10.4%) | 40 (13.1%) | 0.38 |
| Y |  | 420 (87.9%) | 155 (89.6%) | 265 (86.9%) |  |
| CT SCORE, N (%) | 0 |  |  |  | **0.001** |
| 0 |  | 31 (6.5%) | 16 (9.2%) | 15 (4.9%) |  |
| 1 |  | 372 (77.8%) | 144 (83.2%) | 228 (74.8%) |  |
| 2 |  | 46 (9.6%) | 7 (4.0%) | 39 (12.8%) |  |
| 3 |  | 29 (6.1%) | 6 (3.5%) | 23 (7.5%) |  |

**Supplementary Table 4: Patient Demographics and Characteristics Stratified by Survival Status**
Baseline demographic and clinic characteristics of hospitalized patients with COVID-19 (n=478), stratified by survival status (living vs deceased). Continuous variables are presented as mean (SD) and compared using t-test; categorical variables are presented as N (%) and compared using chi-square tests. P-values reflect between-group comparisons.

| **Variable** | **Missing Value** | **Overall**   **(n=478)** | **Living**   **(n=413)** | **Deceased**   **(n=65)** | **Chi-Square or T-test**   **p-value** |
| --- | --- | --- | --- | --- | --- |
| Age (at admission), N | 0 | 478 | 413 | 65 | **<.0001** |
| Mean (SD) |  | 55.7 (15.7) | 53.8 (15.2) | 67.9 (12.7) |  |
| Sex, N (%) | 0 |  |  |  | 0.99 |
| Female |  | 191 (40.0%) | 165 (40.0%) | 26 (40.0%) |  |
| Male |  | 287 (60.0%) | 248 (60.0%) | 39 (60.0%) |  |
| Race, N (%) | 0 |  |  |  | **0.002** |
| Black |  | 183 (38.3%) | 153 (37.0%) | 30 (46.2%) |  |
| Hispanic |  | 127 (26.6%) | 116 (28.1%) | 11 (16.9%) |  |
| Other |  | 107 (22.4%) | 99 (24%) | 8 (12.3%) |  |
| White |  | 61 (12.8%) | 45 (10.9%) | 16 (24.6%) |  |
| BMI, N | 6 | 472 | 407 | 65 | **0.006** |
| Mean (SD) |  | 30.6 (8.1) | 31.0 (8.1) | 28.1 (7.4) |  |
| Obesity, N (%) | 0 |  |  |  | 0.17 |
| N |  | 264 (55.2%) | 223 (54.0%) | 41 (63.1%) |  |
| Y |  | 214 (44.8%) | 190 (46.0%) | 24 (36.9%) |  |
| Weight, N | 5 | 473 | 408 | 65 | **0.006** |
| Mean (SD) |  | 89.5 (45.7) | 90.9 (48.2) | 80.5 (23.0) |  |
| Length of Stay (in Days), N | 0 |  |  |  | **0.0001** |
| Mean (SD) |  | 7.5 (8.3) | 6.5 (6.3) | 14.0 (14.5) |  |
| ICU_hours, N | 6 | 472 | 411 | 61 | **0.0005** |
| Mean (SD) |  | 31.5 (97.5) | 20.4 (72.6) | 106.2 (179.0) |  |
| **Supplementary Table 5: Diabetes-Related Characteristic Stratified by Survival Status**  Diabetes-related clinical features, glycemic indices, pharmacologic therapies, and steroid exposure among hospitalized patients with COVID-19, stratified by survival status. Admission and longitudinal HbA1c values, insulin use, hyperglycemia, steroid-induced hyperglycemia, and inpatient steroid dosing are presented. Continuous variables are shown as mean (SD); categorical variables as N (%). | | | | | |
| **Variable** | **Missing Value** | **Overall**   **(n=478)** | **Living**   **(n=413)** | **Deceased**   **(n=65)** | **Chi-Square or T-test**   **p-value** |
| Admission HbA1c, N (%) | 0 |  |  |  | 0.70 |
| 6.5-7.9 |  | 49 (10.3%) | 40 (9.7%) | 9 (13.8%) |  |
| <=6.4 |  | 52 (10.9%) | 46 (11.1%) | 6 (9.2%) |  |
| >=8.0 |  | 52 (10.9%) | 44 (10.7%) | 8 (12.3%) |  |
| Glucose , N (%) | 0 |  |  |  | 0.24 |
| 200-300 mg/dL  11.1-16.7 mmol/L |  | 42 (8.8%) | 35 (8.8%) | 7 (10.8%) |  |
| <200 mg/dL  <11.1 mmol/L |  | 403 (84.3%) | 353 (85.5%) | 50 (76.9%) |  |
| >300 mg/dL  >16.7 mmol/L |  | 33 (6.9%) | 25 (6.1%) | 8 (12.3%) |  |
| Length of Stay (in Days), N | 0 |  |  |  | **0.0001** |
| Mean (SD) |  | 7.5 (8.3) | 6.5 (6.3) | 14.0 (14.5) |  |
| ICU hours, N | 6 | 472 | 411 | 61 | **0.0005** |
| Mean (SD) |  | 31.5 (97.5) | 20.4 (72.6) | 106.2 (179.0) |  |
| Diabetes Diagnosis, N (%) | 0 |  |  |  | **0.009** |
| N |  | 305 (63.8%) | 273 (66.1%) | 32 (49.2%) |  |
| Y |  | 173 (36.2%) | 140 (33.9%) | 33 (50.8%) |  |
| T2D, N (%) | 0 | 174 (36.4%) | 137 (33.2%) | 37 (56.9%) |  |
| T1D, N (%) |  | 4 (0.8%) | 4 (1.0%) | 0 (0.0%) |  |
| Prediabetes, N (%) | 0 |  |  |  | 0.33 |
| N |  | 451 (94.4%) | 388 (93.9%) | 63 (96.9%) |  |
| Y |  | 27 (5.6%) | 25 (6.1%) | 2 (3.1%) |  |
| Admission HbA1c, N | 325 | 153 | 130 | 23 | 0.86 |
| Mean (SD) |  | 7.9 (2.4) | 7.9 (2.4) | 8.0 (2.7) |  |
| Post-discharge  HbA1c |  |  |  |  |  |
| HbA1c 3m , N | 446 | 32 | 29 | 3 | 0.11 |
| Mean (SD) |  | 6.9 (1.7) | 6.8 (1.8) | 7.4 (0.2) |  |
| HbA1c 3-6m, N | 444 | 34 | 29 | 5 | 0.54 |
| Mean (SD) |  | 7.1 (1.8) | 7.2 (1.9) | 6.6 (0.9) |  |
| HbA1c 6-12m, N | 431 | 47 | 44 | 3 | 0.41 |
| Mean (SD) |  | 7.5 (2.3) | 7.5 (2.3) | 6.4 (1.5) |  |
| HbA1c 12-18m, N | 426 | 52 | 49 | 3 | 0.57 |
| Mean (SD) |  | 6.9 (1.9) | 6.9 (2.0) | 6.3 (1.5) |  |
| HA1c >18m, N | 424 | 54 | 53 | 1 |  |
| Mean (SD) |  | 6.9 (2.0) | 6.9 (2.0) | 5.7 (.) |  |
| Metformin, N (%) | 0 |  |  |  | 0.78 |
| N |  | 431 (90.2%) | 373 (90.3%) | 58 (89.2%) |  |
| Y |  | 47 (9.8%) | 40 (9.7%) | 7 (10.8%) |  |
| DPP4I, N (%) | 0 |  |  |  | 0.24 |
| N |  | 455 (95.2%) | 395 (95.6%) | 60 (92.3%) |  |
| Y |  | 23 (4.8%) | 18 (4.4%) | 5 (7.7%) |  |
| Insulin, N (%) | 0 |  |  |  | **<.0001** |
| N |  | 283 (59.2%) | 259 (62.7%) | 24 (36.9%) |  |
| Y |  | 195 (40.8%) | 154 (37.3%) | 41 (63.1%) |  |
| GLP1RA, N (%) | 0 |  |  |  | 0.57 |
| N |  | 476 (99.6%) | 411 (99.5%) | 65 (100.0%) |  |
| Y |  | 2 (0.4%) | 2 (0.5%) | 0 (0.0%) |  |
| Hyperglycemia, N (%) | 0 |  |  |  | **0.002** |
| No |  | 316 (66.1%) | 284 (68.8%) | 32 (49.2%) |  |
| Yes |  | 162 (33.9%) | 129 (31.2%) | 33 (50.8%) |  |
| Steroid-induced hyperglycemia, N (%) | 0 |  |  |  | **0.007** |
| N |  | 443 (92.7%) | 388 (93.9%) | 55 (84.6%) |  |
| Y |  | 35 (7.3%) | 25 (6.1%) | 10 (15.4%) |  |
| Post Transplant DM, N (%) | 0 |  |  |  | 0.69 |
| N |  | 477 (99.8%) | 412 (99.8%) | 65 (100.0%) |  |
| Y |  | 1 (0.2%) | 1 (0.2%) | 0 (0.0%) |  |
| New Onset Diabetes, N (%) | 0 |  |  |  | 0.85 |
| N |  | 458 (95.8%) | 396 (95.9%) | 62 (95.4%) |  |
| Y |  | 20 (4.2%) | 17 (4.1%) | 3 (4.6%) |  |
| Steroids Received, N (%) | 0 |  |  |  | 0.17 |
| N |  | 129 (27.0%) | 116 (28.1%) | 13 (20.0%) |  |
| Y |  | 349 (73.0%) | 297 (71.9%) | 52 (80.0%) |  |
| MethylPrednisone, N (%) | 0 |  |  |  | **<.0001** |
| N |  | 248 (51.9%) | 232 (56.2%) | 16 (24.6%) |  |
| Y |  | 230 (48.1%) | 181 (43.8%) | 49 (75.4%) |  |
| Initial Total daily Steroid dose, N | 0 | 478 | 413 | 65 | **0.005** |
| Mean (SD) |  | 49.9 (100.0) | 42.0 (84.0) | 100.6 (161.3) |  |

**Supplementary Table 6: Patient Comorbidities Stratified by Living Status**

Pre-existing comorbid conditions, inflammatory biomarkers, respiratory support requirements, consult services, and readmission outcomes among patients hospitalized with COVID-19, stratified by survival status. Comparisons between living and deceased patients were performed using chi-square or t-tests as appropriate.

| **Variable** | **Missing Value** | **Overall**   **(n=478)** | **Living**   **(n=413)** | **Deceased**   **(n=65)** | **Chi-Square or T-test**   **p-value** |
| --- | --- | --- | --- | --- | --- |
| Microvascular complications, N (%) | 0 |  |  |  | **0.005** |
| N |  | 424 (88.7%) | 373 (90.3%) | 51 (78.5%) |  |
| Y |  | 54 (11.3%) | 40 (9.7%) | 14 (21.5%) |  |
| Macrovascular complications, N (%) | 0 |  |  |  | **0.0001** |
| N |  | 435 (91.0%) | 384 (93.0%) | 51 (78.5%) |  |
| Y |  | 43 (9.0%) | 29 (7.0%) | 14 (21.5%) |  |
| DKA, N (%) | 0 |  |  |  | 0.34 |
| N |  | 470 (98.3%) | 407 (98.5%) | 63 (96.9%) |  |
| Y |  | 8 (1.7%) | 6 (1.5%) | 2 (3.1%) |  |
| Dyslipidemia, N (%) | 0 |  |  |  | **0.020** |
| N |  | 338 (70.7%) | 300 (72.6%) | 38 (58.5%) |  |
| Y |  | 140 (29.3%) | 113 (27.4%) | 27 (41.5%) |  |
| NAFLD, N (%) | 0 |  |  |  | **0.002** |
| N |  | 473 (99.0%) | 411 (99.5%) | 62 (95.4%) |  |
| Y |  | 5 (1.0%) | 2 (0.5%) | 3 (4.6%) |  |
| Interstitial Lung disease, N (%) | 0 |  |  |  | **0.0002** |
| N |  | 434 (90.8%) | 383 (92.7%) | 51 (78.5%) |  |
| Y |  | 44 (9.2%) | 30 (7.3%) | 14 (21.5%) |  |
| COPD, N (%) | 0 |  |  |  | **<.0001** |
| N |  | 425 (88.9%) | 378 (91.5%) | 47 (72.3%) |  |
| Y |  | 53 (11.1%) | 35 (8.5%) | 18 (27.7%) |  |
| HTN, N (%) | 0 |  |  |  | **0.0006** |
| N |  | 212 (44.4%) | 196 (47.5%) | 16 (24.6%) |  |
| Y |  | 266 (55.6%) | 217 (52.5%) | 49 (75.4%) |  |
| HF, N (%) | 0 |  |  |  | **0.0005** |
| N |  | 412 (86.2%) | 365 (88.4%) | 47 (72.3%) |  |
| Y |  | 66 (13.8%) | 48 (11.6%) | 18 (27.7%) |  |
| Cancer History, N (%) | 0 |  |  |  | **0.002** |
| N |  | 447 (93.5%) | 392 (94.9%) | 55 (84.6%) |  |
| Y |  | 31 (6.5%) | 21 (5.1%) | 10 (15.4%) |  |
| CKD, N (%) | 0 |  |  |  | **<.0001** |
| N |  | 386 (80.8%) | 351 (85.0%) | 35 (53.8%) |  |
| Y |  | 92 (19.2%) | 62 (15.0%) | 30 (46.2%) |  |
| HIV, N (%) | 0 |  |  |  | 0.83 |
| N |  | 469 (98.1%) | 405 (98.1%) | 64 (98.5%) |  |
| Y |  | 9 (1.9%) | 8 (1.9%) | 1 (1.5%) |  |
| ERSD on Dialysis, N (%) | 0 |  |  |  | **0.0004** |
| N |  | 446 (93.3%) | 392 (94.9%) | 54 (83.1%) |  |
| Y |  | 32 (6.7%) | 21 (5.1%) | 11 (16.9%) |  |
| Black, eGFR, N | 11 | 467 | 402 | 65 | **<.0001** |
| Mean (SD) |  | 53.1 (19.6) | 54.9 (18.8) | 41.8 (20.7) |  |
| Non-Black eGFR, N | 12 | 466 | 401 | 65 | **<.0001** |
| Mean (SD) |  | 50.9 (17.1) | 52.8 (15.5) | 38.9 (21.2) |  |
| Transplant patient, N (%) | 0 |  |  |  | **<.0001** |
| N |  | 463 (96.9%) | 407 (98.5%) | 56 (86.2%) |  |
| Y |  | 15 (3.1%) | 6 (1.5%) | 9 (13.8%) |  |
| Asthma, N (%) | 0 |  |  |  | 0.49 |
| N |  | 438 (91.6%) | 377 (91.3%) | 61 (93.8%) |  |
| Y |  | 40 (8.4%) | 36 (8.7%) | 4 (6.2%) |  |
| Sleep Apnea, N (%) | 0 |  |  |  | 0.44 |
| N |  | 451 (94.4%) | 391 (94.7%) | 60 (92.3%) |  |
| Y |  | 27 (5.6%) | 22 (5.3%) | 5 (7.7%) |  |
| Endo consult, N (%) | 0 |  |  |  | **0.0005** |
| N |  | 417 (87.2%) | 369 (89.3%) | 48 (73.8%) |  |
| Y |  | 420 (87.9%) | 364 (88.1%) | 56 (86.2%) |  |
| Endocrine consult, N (%) | 0 |  |  |  | **0.0005** |
| N |  | 417 (87.2%) | 369 (89.3%) | 48 (73.8%) |  |
| Y |  | 61 (12.8%) | 44 (10.7%) | 17 (26.2%) |  |
| Rheum Consult (Y/N), N (%) | 0 |  |  |  | **<.0001** |
| N |  | 392 (82.0%) | 352 (85.2%) | 40 (61.5%) |  |
| Y |  | 86 (18.0%) | 61 (14.8%) | 25 (38.5%) |  |
| Cytokine Storm (Y/N), N (%) | 0 |  |  |  | **<.0001** |
| N |  | 411 (86.0%) | 366 (88.6%) | 45 (69.2%) |  |
| Y |  | 67 (14.0%) | 47 (11.4%) | 20 (30.8%) |  |
| Smoker Status, N (%) | 0 |  |  |  | **<.0001** |
| Current Smoker |  | 64 (13.4%) | 58 (14.0%) | 6 (9.2%) |  |
| Former Smoker |  | 109 (22.8%) | 79 (19.1%) | 30 (46.2%) |  |
| Never Smoker |  | 271 (56.7%) | 252 (61.0%) | 19 (29.2%) |  |
| Unknown |  | 34 (7.1%) | 24 (5.8%) | 10 (15.4%) |  |
| CRP, N | 50 | 428 | 371 | 57 | 0.27 |
| Mean (SD) |  | 13.0 (27.6) | 12.5 (27.7) | 16.8 (27.1) |  |
| D-Dimer combined, N | 32 | 446 | 388 | 58 | **0.097** |
| Mean (SD) |  | 2.9 (10.0) | 2.5 (8.9) | 5.9 (15.2) |  |
| Ferritin, N | 59 | 419 | 366 | 53 | 0.33 |
| Mean (SD) |  | 844.0 (2353.3) | 820.0 (2486.4) | 1009.6 (1045.1) |  |
| IL6, N | 256 | 222 | 192 | 30 | 0.91 |
| Mean (SD) |  | 267.2 (2829.8) | 271.1 (3030.5) | 242.3 (733.3) |  |
| Creatinine kinase, N | 14 | 464 | 402 | 62 | 0.24 |
| Mean (SD) |  | 5.0 (38.9) | 3.2 (23.7) | 16.6 (87.4) |  |
| Readmit for COVID, N (%) | 0 |  |  |  | 0.55 |
| N |  | 449 (93.9%) | 389 (94.2%) | 60 (92.3%) |  |
| Y |  | 29 (6.1%) | 24 (5.8%) | 5 (7.7%) |  |
| Readmit for Non-COVID, N (%) | 0 |  |  |  | 0.95 |
| N |  | 369 (77.2%) | 319 (77.2%) | 50 (76.9%) |  |
| Y |  | 109 (22.8%) | 94 (22.8%) | 15 (23.1%) |  |
| O2 Intervention, N (%) | 0 |  |  |  | **<.0001** |
| BIPAP |  | 4 (0.8%) | 0 (0.0%) | 4 (6.2%) |  |
| BIPAP/HFNC |  | 1 (0.2%) | 1 (0.2%) | 0 (0.0%) |  |
| CPAP |  | 1 (0.2%) | 1 (0.2%) | 0 (0.0%) |  |
| HFNC |  | 40 (8.4%) | 30 (7.3%) | 10 (15.4%) |  |
| HFNC, BIPAP, NRB |  | 1 (0.2%) | 1 (0.2%) | 0 (0.0%) |  |
| HFNT |  | 1 (0.2%) | 1 (0.2%) | 0 (0.0%) |  |
| NC |  | 111 (23.2%) | 99 (24.0%) | 12 (18.5%) |  |
| NRB |  | 12 (2.5%) | 10 (2.4%) | 2 (3.1%) |  |
| RA |  | 284 (59.4%) | 264 (63.9%) | 20 (30.8%) |  |
| VENT |  | 23 (4.8%) | 6 (1.5%) | 17 (26.2%) |  |

**Supplementary Table 8: Patient’s COVID-19 Confirmation Stratified by Survival Status**
Laboratory confirmation of SARS-CoV-2 infection by PCR testing among hospitalized patients, stratified by survival status. Values are presented as N (%) with between-group comparisons using chi-square testing.

| **Variable** | **Missing Value** | **Overall**   **(n=478)** | **Living**   **(n=413)** | **Deceased**   **(n=65)** | **Chi-Square or T-test**   **p-value** |
| --- | --- | --- | --- | --- | --- |
| COVID SWAB POSITIVE (Y/N), N (%) | 0 |  |  |  | 0.65 |
| N |  | 58 (12.1%) | 49 (11.9%) | 9 (13.8%) |  |
| Mean (SD) |  | 31.5 (97.5) | 20.4 (72.6) | 106.2 (179.0) |  |
